# Structural and Mechanistic Insights into a Novel Monooxygenase for Poly(acrylic acid) Biodegradation

**DOI:** 10.3390/ijms25168871

**Published:** 2024-08-15

**Authors:** Rui Feng, Juyi Zhao, Xiaochen Li, Sijun Dong, Dan Ma

**Affiliations:** College of Life Sciences, Hebei Basic Science Center for Biotic Interaction, Hebei University, Baoding 071002, China; 13653278516@163.com (R.F.); 18713417798@163.com (J.Z.); l17861828703@163.com (X.L.); sjdong@iue.ac.cn (S.D.)

**Keywords:** poly(acrylic acid), monooxygenase, polyacrylamide, biodegradation, crystal structure, molecular mechanism

## Abstract

Polyacrylamide (PAM) is a high-molecular-weight polymer with extensive applications. However, the inefficient natural degradation of PAM results in environmental accumulation of the polymer. Biodegradation is an environmentally friendly approach in the field of PAM treatment. The first phase of PAM biodegradation is the deamination of PAM, forming the product poly(acrylic acid) (PAA). The second phase of PAM biodegradation involves the cleavage of PAA into small molecules, which is a crucial step in the degradation pathway of PAM. However, the enzyme that catalyzes the degradation of PAA and the molecular mechanism remain unclear. Here, a novel monooxygenase PCX02514 is identified as the key enzyme for PAA degradation. Through biochemical experiments, the monooxygenase PCX02514 oxidizes PAA with the participation of NADPH, causing the cleavage of carbon chains and a decrease in the molecular weight of PAA. In addition, the crystal structure of the monooxygenase PCX02514 is solved at a resolution of 1.97 Å. The active pocket is in a long cavity that extends from the C-terminus of the TIM barrel to the protein surface and exhibits positive electrostatic potential, thereby causing the migration of oxygen-negative ions into the active pocket and facilitating the reaction between the substrates and monooxygenase PCX02514. Moreover, Arg10-Arg125-Ser186-Arg187-His253 are proposed as potential active sites in monooxygenase PCX02514. Our research characterizes the molecular mechanism of this monooxygenase, providing a theoretical basis and valuable tools for PAM bioremediation.

## 1. Introduction

Polyacrylamide (PAM) is a high-molecular-weight (MW) polymer that is commonly used in oil exploitation, sludge treatment, and agricultural production owing to its excellent mechanical performance, elasticity, flocculation, biocompatibility, and even electric qualities [1,2]. Global annual production of PAM is estimated at 9 MT/year and is growing rapidly [3]. However, the relatively high MW of PAM limits its degradation efficiency, resulting in its environmental accumulation and causing ecosystem disruption [4]. For instance, a decline in soil porosity has been reported when the amount of PAM exceeds 0.09 g kg^−1^, which leads to a decrease in oxygen supply to the soil and impedes the growth of plants [5]. Therefore, the treatment of PAM is attracting attention. For PAM degradation, physical or chemical methods, including oxidative, photooxidative, thermal degradation, UV radiation, pyrolysis, and mechanical approaches, have some drawbacks, such as secondary pollution problems and considerable financial costs [6,7]. Hence, using renewable biological entities (i.e., enzymes or microorganisms) for biodegradation is an ideal and environmentally friendly approach [8].

Many species of bacteria have been isolated for PAM degradation, including *Bacillus cereus*, *Bacillus flexus* [9], *Acinetobacter* sp., *Bacillus sphaericus* [10], *B. cereus* strain PM-2, *Bacillus* sp. PM-3 [11], and *Klebsiella* sp. PCX [5]. However, the complete metabolic pathways of PAM degradation remain unclear [12]. Previous research has reported that the first phase of PAM biodegradation is the deamination of PAM, catalyzed by amidohydrolase [13]. This process produces poly(acrylic acid) (PAA), another high-MW polymer [14]. The second phase of PAM degradation involves oxidizing the carbon chains in PAA [15]. During this process, the main carbon backbone is cleaved, and the PAA molecules are gradually transformed into small molecules. These small molecules can be further decomposed by bacteria, providing carbon and nitrogen sources for cell growth, and are ultimately degrading into fully reduced or oxidized products (CO_2_ and H_2_O) [12]. Therefore, the cleavage of polymers into small molecules is a crucial step in the degradation pathway of PAM [12]. However, the products of PAA oxidation and the mode of carbon chain cleavage during oxidation have yet to be elucidated. 

Monooxygenases are enzymes that catalyze the insertion of a single oxygen atom from O_2_ into an organic substrate [16]. Molecular oxygen accepts electrons from the coenzymes NAD(P)H and is then activated to initiate the oxygenation of the organic substrate [17]. Based on the type of cofactors required for catalysis, monooxygenases are classified into seven families: heme-dependent monooxygenases, flavin-dependent monooxygenases, copper-dependent monooxygenases, non-heme iron-dependent monooxygenases, pterin-dependent monooxygenases, other cofactor-dependent monooxygenases, and cofactor-independent monooxygenases [16]. The monooxygenases in each family are further divided into several subclasses, catalyzing various oxidation reactions of different substrates. These reactions include hydroxylation, epoxidation, Baeyer–Villiger oxidation, heteroatom dealkylation, dehalogenation, dehydrogenation, dehydration, and reduction [18]. For instance, cytochrome P450 monooxygenase from *Bacillus megaterium* BM3 can hydroxylate various alkanes, fatty acids, and aromatic compounds [19]. Therefore, monooxygenase may be the key enzyme for PAA degradation [12]. However, direct evidence for the degradation of PAA by monooxygenases is lacking, and the precise binding mode and underlying mechanism between monooxygenases and PAA remain unclear. 

*Klebsiella* sp. PCX is a newly isolated gram-negative bacterium with excellent potential for PAM degradation [5,20], as its degradation period (within 48 h) is much shorter than that reported for other bacteria (up to 14 days [21]). This study identified a novel monooxygenase PCX02514 from the genome of *Klebsiella* sp. PCX, confirming its ability for PAA degradation. In addition, the structure of monooxygenase PCX02514 was solved, and its active sites were validated. Collectively, the mechanism through which monooxygenase PCX02514 catalyzes PAA was proposed. This study provides a theoretical basis and enriches the enzyme resources available for PAM degradation.

## 2. Results and Discussion

### 2.1. Bioinformatic Analyses of Monooxygenase PCX02514 with PAA Degradation Ability

**Figure 1:**
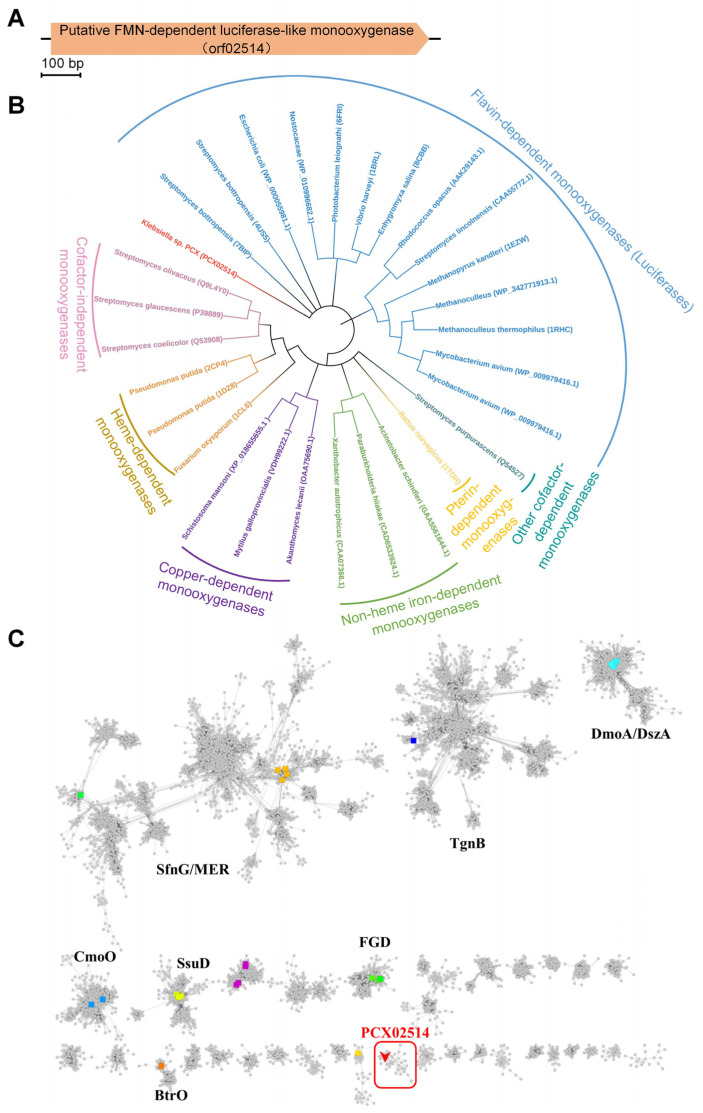
Bioinformatic analyses of monooxygenase PCX02514 with PAA degradation activity. (**A**) The gene (orf02514) encoding the monooxygenase PCX02514. (**B**) Phylogenetic tree of monooxygenases. (**C**) Sequence similarity network of the luciferase-like domain. UniProt accession numbers: SfnG—FMNH2-dependent dimethylsulfone monooxygenase (Q65YW9, green); MER—5,10-methylenetetrahydromethanopterin reductase (O29196, Q50744, dark yellow); TgnB—flavin-dependent trigonelline monooxygenase (Q6F9F6, dark blue); DmoA—dimethyl-sulfide monooxygenase (E9JFX9, cyan); DszA—dibenzothiophene-sulfone monooxygenase (P54995, cyan); CmoO—N-acetyl-S-alkylcysteine monooxygenase (O34846, blue); SsuD—alkanesulfonate monooxygenase (P40402, yellow); FGD—F420-dependent glucose-6-phosphate dehydrogenase (D6ZA79, A0QQJ4, light green); BtrO—4-(Gamma-L-glutamylamino) butanoyl-[Btrl acyl-carrier protein] monooxygenase (Q4H4E5, orange). Monooxygenase PCX02514 is highlighted in the red box.

Bacterial luciferase is a flavin monooxygenase that catalyzes the oxidation of a long-chain aldehyde [22]. Owing to the degradation of PAA being caused by oxidation reactions and PAA having a long-chain structure [23], we speculated that bacterial luciferase might be involved in the degradation of PAA. In this study, according to the genome sequencing data of *Klebsiella* sp. PCX (GenBank accession no. CP091527.1), a gene encoding a putative FMN-dependent luciferase-like monooxygenase (orf02514, accession no. UKB70026.1), was identified (Figure 1A). Multiple sequences of monooxygenases were collected and used to construct a phylogenetic tree (Figure 1B). Phylogenetic analysis revealed that the monooxygenases were divided into seven classes, among which PCX02514 was clustered on an individual branch and was closely related to luciferases. These findings indicate that monooxygenase PCX02514 might be an unclassified monooxygenase and able to catalyze long-chain substrates. To determine the specificity of PCX02514, a sequence similarity network (SSN) of the luciferase-like domain (Pfam accession no. IPR011251) was performed. The sequences of the luciferase-like domain exhibited diversity and were divided into distinct clusters based on different catalytic activities (Figure 1C). The most closely related proteins were clustered together and shared the same function [24]. Monooxygenase PCX02514 was separated from other characteristic proteins and was located in a distinct cluster where the function and structure of the proteins had not been characterized and solved. Consequently, the monooxygenase PCX02514 identified in this work is a novel enzyme whose function needs to be further determined. 

### 2.2. Biochemical Analyses of Monooxygenase PCX02514

**Figure 2:**
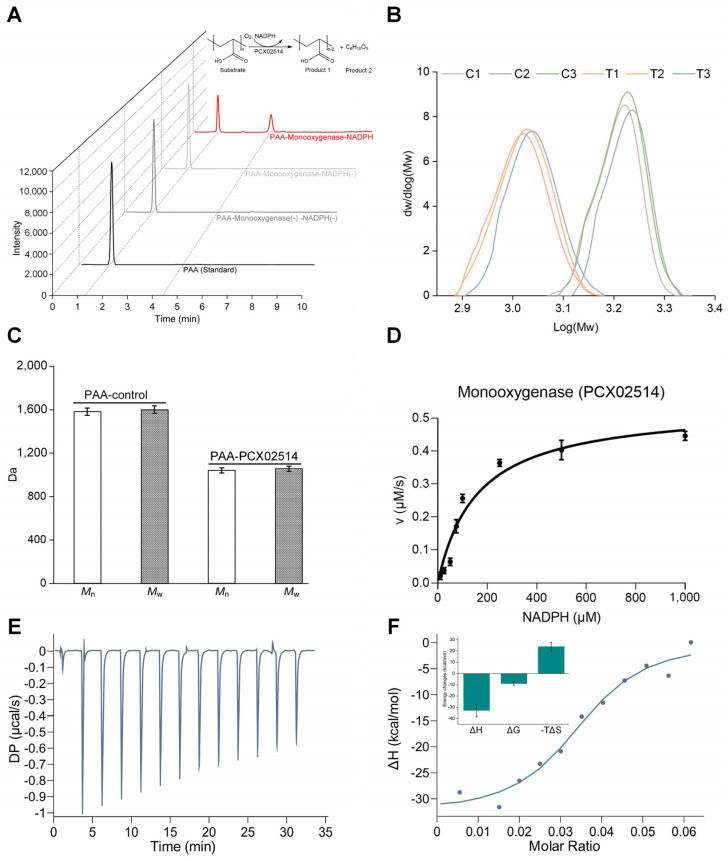
Biochemical analyses of monooxygenase PCX02514. (**A**) HPLC–MS elution chromatograms for PAA–PCX02514–NADPH. (**B**) Molecular weight distribution curves of PAA. C1, C2, and C3: PAA–control group; T1, T2, T3: PAA–PCX02514 group. (**C**) Molecular weight of PAA in the PAA–control group and PAA–PCX02514 group. (**D**) Enzyme kinetic assay for monooxygenase PCX02514. (**E**,**F**) ITC profiles of the interaction between PAA and PCX02514. PAA: 120 μM, PCX02514: 40 μM, Buffer: PBS (pH 7.4, containing 100 μM NADPH). The inlet of (**F**) reflects the changes in enthalpy (ΔH,) Gibbs free energy (ΔG), and entropy (ΔS).

For the functional analysis of the newly identified monooxygenase, heterologous expression was performed, and the purified monooxygenase PCX02514 yielded a single band with a molecular mass of approximately 35 kDa in sodium dodecyl sulfate-polyacrylamide gel electrophoresis (SDS-PAGE) (Figure S1). Cleavage of PAA carbon chains was previously proposed to be oxidized by monooxygenase, thereby achieving the goal of PAA degradation [23]. However, no evidence of the activity of monooxygenase had been offered in previous research. In this study, the products obtained after degradation by monooxygenase PCX02514 were detected using high-performance liquid chromatography–mass spectrometry (HPLC–MS) (Figure 2A and Figure S2, and Table S1). Most monooxygenases require NAD(P)H cofactors for electron transfer, where oxygen molecules are activated to complete the catalysis process of substrate oxidation [16]. Similar results were observed in this study, with the oxidation of PAA occurring only in the presence of NADPH. Negative controls omitting either monooxygenase PCX02514 or NADPH did not yield any products, and only the parent substance PAA was detected. Moreover, other cofactors, including NADH, FAD, FMN, and FMNH_2_, could not complete the catalytic reactions. These properties of monooxygenase PCX02514 differ from those of bacterial luciferases, which employ reduced flavin as a substrate rather than as a cofactor [22]. In HPLC, a peak with a retention time of 4.350 min appeared after the degradation of PAA by PCX02514 with NADPH. Mass spectrometry identified the molecule associated with this peak as C_6_H_10_O_5_. Previous research hypothesized that the aerobic reaction of PAA was similar to the α-oxidation of fatty acids, with the α-carbon of PAA being oxidized to -COH, resulting in the cleavage of carbon chains [11]. In contrast, Kawai (1994) assumed that the degradation of PAA is similar to the β-oxidation of fatty acids, with PAA generating a β-hydroxyl group after oxidation [25]. In addition, there might be other mechanisms involved in the formation of the product C_6_H_10_O_5_. The mass spectra of oligo acrylic acid agreed with a hydrogen-initiated group, which is often produced via chain transfer reactions, and an end group with C=C double bond moieties produced through disproportion. Another possible mechanism for the formation of the hydrogen end group involves the decarboxylation of the carboxylic acid moieties produced via oxidative degradation, which implies degradation initiated from the hydrogen end group. Additionally, the product C_6_H_10_O_5_ agreed with the dimer of acrylic acid with H and OH ends, which might be produced via hydrolysis at the carbon-carbon bond between the second and third units or through decarboxylative degradation. Our results show that monooxygenase PCX02514 catalyzes the hydroxylation of PAA, leading to the cleavage of the carbon chain, producing C_6_H_10_O_5_, and eventually, long-chain PAA was cleaved into short-chain PAA. Subsequently, the chain length of PAA was characterized (Table S2). The MW distribution graph showed significant differences between the PAA–control group and the PAA–PCX02514 group (Figure 2B). The biodegradation of PAA involves enzymatic reactions that have been reported to alter the physical and chemical properties of polymers [11], and low MW products are produced through the biodegradation of PAA [26]. Similarly, compared with the control group, the MW values (i.e., *M*_n_ and *M*_w_) of PAA in the PAA–PCX02514 group decreased by 34.26% and 34.01%, respectively (Figure 2C), indicating that the long-chain structure of PAA was depolymerized into lower MW fragments. These observations further supported the depolymerization of PAA. 

Next, the initial reaction rate against NADPH concentration was used to fit the Michaelis–Menten curve (Figure 2D). The turnover rate (*k_cat_*) and the Michaelis constant (*K_M_*) of PCX02514 were 0.00214 s^−1^ and 158.9 μM, respectively (Table 1), providing further evidence that monooxygenase PCX02514 could catalyze PAA. Isothermal titration calorimetry (ITC) experiments were conducted to determine the thermodynamic characteristics of the binding between monooxygenase PCX02514 and PAA (Figure 2E,F). The ITC results showed that this binding interaction was a spontaneous and exothermic process, with stable compounds formed after binding. The major thermodynamic parameters related to PAA–PCX02514 binding are shown in Table 2. For strong ligand–protein interactions, the values of binding constants are normally more than 10^7^ M^−1^ [27]. The binding constant value between monooxygenase PCX02514 and PAA was 10^11^ M^−1^, which indicated rapid diffusion of PAA to the target site of PCX02514. The ITC results suggested that monooxygenase PCX02514 binds specifically to PAA, with hydrogen bonds and van der Waals forces acting as the driving force. In summary, monooxygenase PCX02514, responsible for PAA degradation, was identified and verified through biochemical experiments in this study. 

### 2.3. Crystal Structure of Monooxygenase PCX02514

**Figure 3:**
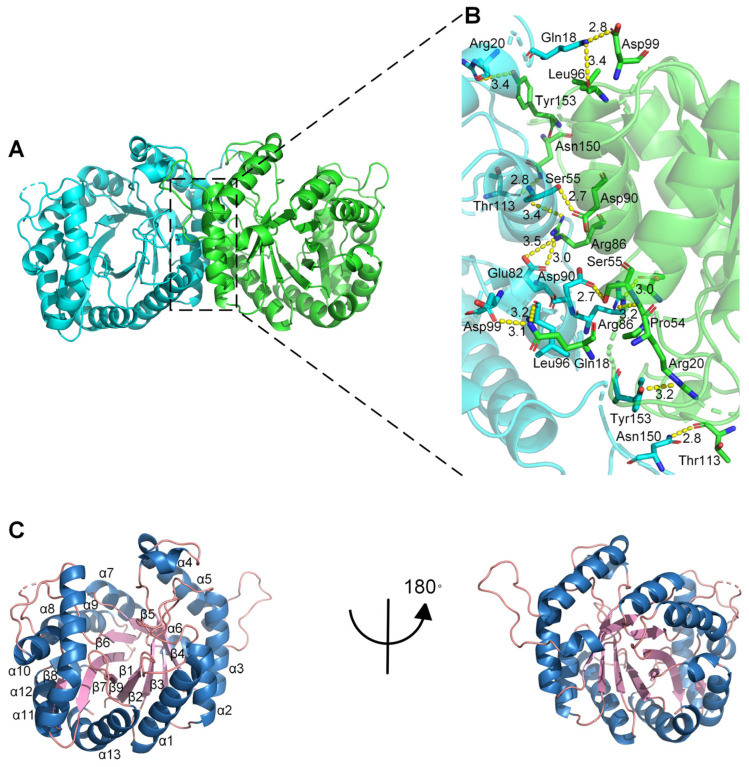
Overall structure of monooxygenase PCX02514. (**A**) Cartoon diagram showing the overall structure of monooxygenase PCX02514. Chain A and chain B of the dimer are shown in cyan and green, respectively. (**B**) Stereo view of the ribbon–stick model showing the detailed binding interface between chain A and chain B. Bonds involved in the interactions are shown as dotted lines. (**C**) Cartoon diagram showing the monomer structure of monooxygenase PCX02514. The α-helix, β-sheet, and loops are shown in blue, light pink, and salmon, respectively.

To explore the catalytic mechanism of the monooxygenase PCX02514, X-ray diffraction was used to solve the crystal structure of PCX02514 at a resolution of 1.97 Å (Protein Data Bank (PDB) ID: 8K74), and the X-ray diffraction pattern is shown in Figure S3. The asymmetric unit of the crystal was composed of two homodimers, whose monomers were related by a crystallographic two-fold symmetry axis that was located parallel to the central strands (Figure 3A), and hydrogen bonds played a crucial role in dimer formation (Figure 3B). The monomer was composed of a single domain with an eight-stranded α/β-barrel (TIM-barrel) fold, and the first structurally elucidated protein with this architecture was triosephosphate isomerase [28]. Except for β8, formed by residues 275–277, eight β-strands formed the inner barrel, which was surrounded by eight α-helices. Each α-helix was connected to a strand through short loop regions, apart from five insertion regions: α4, α6, α10, α11, and α12. The central strands of the (α/β)_8_ barrel in monooxygenase PCX02514 were arranged in a funnel-shaped barrel that broadened toward its C-terminus, thereby creating a large, roughly oval bottom (Figure 3C). Bacterial luciferase is an a-β heterodimer, and the individual subunits fold into a single domain (β/α)_8_ barrel [22]. A TIM barrel core similar to that of PCX02514 was found in LuxAB [22], Mer [29], and SsuD [30], which are members of the bacterial luciferase family that possess the structural characteristics of a TIM barrel. 

By conducting a BLAST search in the PDB database, six members of the bacterial luciferase family with sequence similarity (the highest sequence identity was 38.28%) to the monooxygenase PCX02514 were identified, and their structures were compared. The closest structural homolog to PCX02514 was luciferase-like monooxygenase from *Streptomyces bottropensis* (PDB ID: 7BIP), which could form an epoxide during rishirilide biosynthesis [31], a reaction that was different from the hydroxylation of PAA catalyzed by PCX02514. These two monooxygenases have the same dimer arrangement, and the dimer formation has a general stabilizing function (Figure S4A). The five other members of the bacterial luciferase family that exhibited sequence similarity to PCX02514 had different folds, structure domains, subunits, and polymerization patterns (Figure S4B–F). Owing to significant differences in their structures, the functions of the homologs also differ. For example, bacterial luciferase from *Vibrio harveyi* (PDB ID: 1BRL) catalyzes the oxidation of FMNH_2_ and a long-chain aliphatic aldehyde [32]; F420-dependent alcohol dehydrogenase from *Methanoculleus thermophilus* (PDB ID: 1RHC) could bind acetone or isopropanol oxygens for catalysis [33]; and luciferase-like monooxygenase from *Streptomyces bottropensis* (PDB ID: 4US5) introduces an epoxy group at the end of the biosynthesis of mensacarcin [34]. These results indicate that members of the bacterial luciferase family exhibit diverse sequences and structures and could catalyze various reaction types. 

### 2.4. Active Sites of Monooxygenase PCX02514 Binding with Substrates

**Figure 4:**
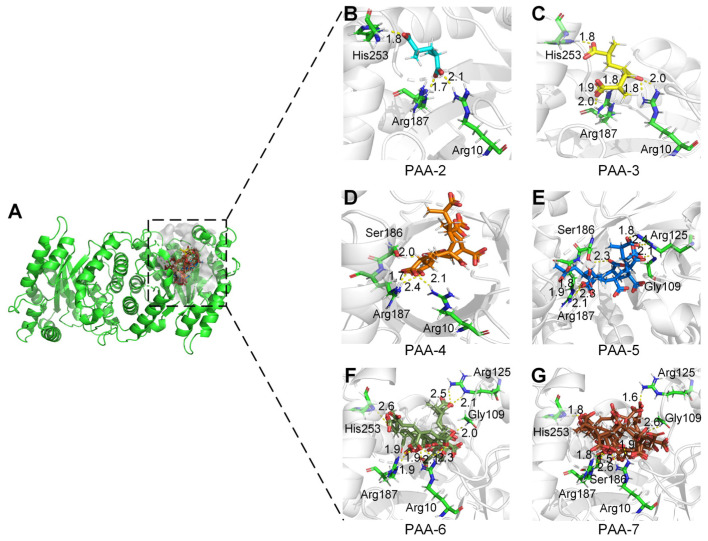
Active sites of monooxygenase PCX02514. (**A**) Active pocket surface model of monooxygenase PCX02514. (**B**–**G**) Enlarged views of the active sites of monooxygenase PCX02514. Residues that interacted with PAAs are shown as green sticks. The bonds involved in the interactions were shown as dotted lines.

To determine the active sites of monooxygenase PCX02514 for PAA degradation, interactions between the substrate and the enzyme were predicted using molecular docking. The active pocket of monooxygenase PCX02514 was in a long cavity that extended from the C-terminus of the TIM barrel to the protein surface, and each monomer in the dimer had an active pocket at the same position (Figure 4A). The substrate-binding tunnel of monooxygenase PCX02514 was open at both ends, which was considered more suitable for accommodating long-chain substrates such as PAA. PAA-2, PAA-3, PAA-4, PAA-5, PAA-6, and PAA-7 formed hydrogen bond interactions with the residues Arg10, Arg125, Ser186, Arg187, and His253 of monooxygenase PCX02514 (Figure 4B–G and Figure S5). Similar structures of homologous monooxygenase were searched in the PDB database. Results showed that the structure of PCX02514 was similar to that of F420-dependent alcohol dehydrogenase from *Methanoculleus thermophilus* (PDB ID: 1RHC), which is a member of the bacterial luciferase family [33]. Comparing the substrate–enzyme interaction networks of these two proteins, we found that their binding pockets were quite similar (Figure S6). Although the substrates were different, the positions of substrates were close, and hydrogen bonds were the primary forces facilitating substrates binding at the active sites of the corresponding receptors. Moreover, a structural conservation analysis of the residues surrounding the binding pocket was analyzed. Results showed that the binding pocket of monooxygenase PCX02514 was conserved, indicating the accuracy of the binding pocket (Figure S7). In addition, the overall electrostatic potential of residues surrounding the active pockets was analyzed (Figure S8), with the results showing that the pocket had a positive electrostatic potential, which is conducive to the migration of oxygen-negative ions into the active pocket. 

**Figure 5:**
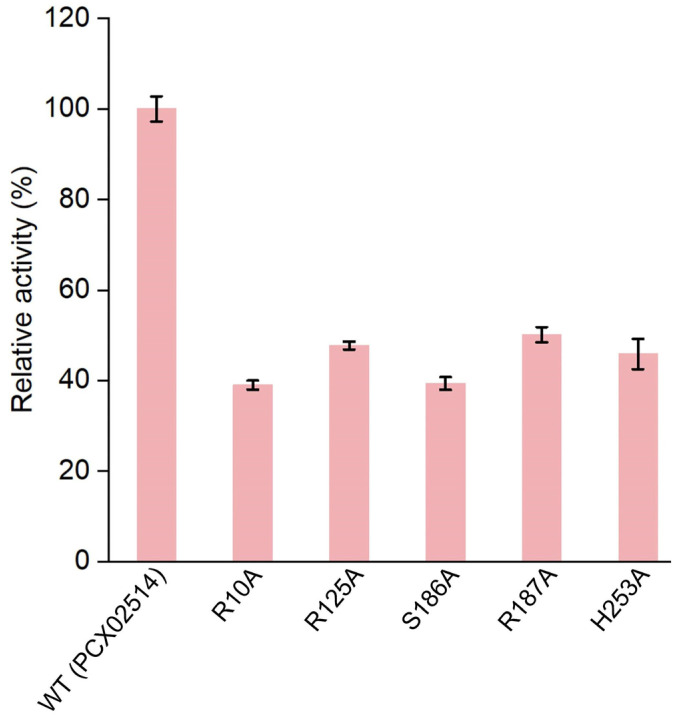
Relative enzyme activities of wild-type (WT) and mutants of monooxygenase.

Based on molecular docking results, key amino acids that interacted with PAAs were identified (i.e., Arg10, Arg125, Ser186, Arg187, and His253). Subsequently, by combining key amino acid mutations, five mutants (R10A, R125A, S186A, R187A, and H253A) were obtained to verify the catalytic sites of monooxygenase PCX02514. The five mutants were expressed and purified (Figure S9), and their relative enzyme activities were measured (Figure 5). Similar to the monooxygenase from *Aspergillus flavus*, the active site arginine is crucial for positioning and guiding the cofactor and substrate during catalysis [35]. In this study, the mutants R10A, R125A, and R187A exhibited a decrease in enzyme activity. This reduced activity might be due to Arg10, Arg125, and Arg187 being alkaline amino acids that attracted multiple carboxyl groups in PAA to move toward the active pocket, ultimately forming hydrogen bonds. However, the functions of the active sites Ser186 and His253 in the catalytic activity of monooxygenase PCX02514 have not previously been reported. Compared with the wild-type monooxygenase PCX02514, the mutant S186A exhibited more than 50% reduced activity, indicating that the mutation of this residue at the active site partially abolished the activity of monooxygenase PCX02514. The active site Ser186 might be related to stabilizing the transition state, thereby promoting the catalytic function of monooxygenase PCX02514. The enzyme activity of mutant H253A also decreased, suggesting that the His253 residue might play an important role in the monooxygenase-mediated catalysis of PAA. The small steric hindrance of His253 means the residue likely promoted the entry of substrates and the release of products. In summary, these results confirm the critical roles of residues Arg10, Arg125, Ser186, Arg187, and His253 in PAA degradation by monooxygenase PCX02514. 

## 3. Materials and Methods

### 3.1. Bacterial Strain, Culture Medium, and Chemicals

The gram-negative bacterium *Klebsiella* sp. PCX (GenBank accession no. CP091527.1) was obtained from the laboratory at Southwest Petroleum University (Chengdu, China) [5,20]. *Escherichia coli* DH5α and *E. coli* BL21 (DE3) (TransGen Biotech, Beijing, China) were used for heterologous expression. All *E. coli* strains were grown aerobically at 37 °C in LB medium (10 g L^−1^ NaCl, 5 g L^−1^ yeast extract, and 10 g L^−1^ tryptone) supplemented with the appropriate antibiotics. The average MW of PAA (Aladdin Co., Ltd., Shanghai, China) was approximately 2000 Da. All other reagents used in this study were obtained from Sigma Co., Ltd. (Shanghai, China). The enzymes used for DNA manipulation were obtained from TaKaRa Biotechnology (Dalian, China).

### 3.2. Bioinformatic Analyses 

A gene (orf02514, accession no. UKB70026.1) annotated as a monooxygenase of the luciferase family in the genome of *Klebsiella* sp. PCX (GenBank accession no. CP091527.1) was identified as potentially involved in the degradation of PAA. Sequences of monooxygenases used to construct the phylogenetic tree were collected from the National Center for Biotechnology Information (NCBI), PDB, and UniProt databases. The phylogenetic tree of monooxygenases was constructed using the neighborhood-joining method in MEGA software (https://www.megasoftware.net/ (Version number: 10.0)). Sequences of the luciferase-like domain (Pfam accession no. IPR011251) with an E-value cut-off of 10^−50^ were used to generate an SSN using the Enzyme Function Initiative-Enzyme Similarity Tool (https://efi.igb.illinois.edu/efi-est/ (Version number: 2024_03/100)) [36], where each node represented a collection of sequences with ≥90% sequence identity. The SSN was visualized using Cytoscape v3.9 [37].

### 3.3. Cloning, Expression, and Purification

The physicochemical properties of monooxygenase PCX02514 were predicted using ProtParam from ExPASy (http://web.expasy.org/protparam/ (accessed on 8 August 2024)) and are shown in Table S3. For the construction of the prokaryotic expression vector, the gene (orf02514) encoding monooxygenase PCX02514 was amplified using PCR (Table S4). The amplification product and the vector pGEX-6P-1 were subjected to *Eco*RI + *Xho*I double digestion, followed by agarose gel electrophoresis. The digested fragments were recovered, ligated with DNA ligase, and transformed into competent *E. coli* DH5α cells. A single colony was selected and cultured at 37 °C for 12 h, and then the plasmid was extracted and identified by sequencing. Subsequently, for protein expression, the recombinant plasmid was transformed into competent *E. coli* BL21(DE3) cells, and a single colony was selected and cultured in a liquid LB medium containing ampicillin at 37 °C until an optical density at 600 nm (OD_600_) of 0.6–0.8 was reached. Isopropyl β-D-1-thiogalactopyranoside at a final concentration of 0.5 mM was used to induce protein expression at 16 °C for 18 h. The bacterial cells were then harvested via centrifugation, resuspended in ice-cold buffer A (50 mM Tris-HCl, pH 8.0), and disrupted through sonication. The resulting lysates were clarified via centrifugation at 15,000 rpm for 30 min. The supernatant was collected and applied to a glutathione S-transferase (GST) column (GenScript, Nanjing, Chian) pre-equilibrated with buffer A, and the GST tag was removed by PreScission Protease, which was purified in our laboratory and stored in buffer B (50 mM Tris, 150 mM NaCl, 10 mM EDTA, 1 mM DTT, and 20% (*v*/*v*) glycerol, pH 8.0) at a concentration of 3 mg mL^−1^. After affinity chromatography, the target protein was loaded onto a HiTrap Q FF column (Cytiva, San José, CA, USA). The column was washed with 5 mL of buffer A, and the protein was eluted with a linear gradient of 0–1 M NaCl in 40 mL of buffer A. Fractions containing the target protein were collected, and buffer C (50 mM Tris-HCl, pH 8.0, and 150 mM NaCl) was used for size exclusion chromatography (HiLoad 16/600 Superdex 200 prep grade, Cytiva, CA, USA). The purified protein was examined using 12% SDS-PAGE with a commercial protein marker (Solarbio, Beijing, China) and visualized by Coomassie blue staining to check its purity. The concentration of the purified protein was measured using a NanoPhotometer N60 (IMPLEN, Munich, Germany). The purified protein was subsequently concentrated to 15 mg mL^−1^ using a centrifugal filter (MWCO 10000, Millipore, MA, USA). Aliquots of the protein were flash-frozen with liquid nitrogen and stored at −80 °C until further use.

### 3.4. HPLC–MS Analysis 

HPLC–MS analysis was performed on a Waters 2695-ZQ2000 HPLC-MS system (Shanghai, China) operating in ion scan mode. A 2 mL reaction mixture containing 500 μM PAA, 500 μM NADPH, 14 μM monooxygenase PCX02514, and 1 × PBS buffer (pH 7.4) was incubated for 5 h at 30 °C. Meanwhile, reactions without NADPH and/or monooxygenase PCX02514 were set as controls. All samples were filtered through 0.22 μm membranes and then analyzed using the HPLC–MS system that was equipped with a Spherisorb ODS-1 C18 column (4.6 × 150 mm, 5 μm). The mobile phase was 0.025 mol L^−1^ KH_2_PO_4_ and 0.025 mol L^−1^ Na_2_HPO_4_ (pH 6.86). The flow rate was 1.0 mL min^-1^ with an injection volume of 20 μL, and the effluent was monitored at a wavelength of 254 nm. Mass spectrometric analysis was performed in data-dependent acquisition mode, and survey scans were obtained in a mass range of 80–1000 *m*/*z*.

### 3.5. Gel Permeation Chromatography Analysis

The reaction mixture for the treatment group, containing 500 μM PAA, 500 μM NADPH, 14 µM monooxygenase PCX02514, and 1 × PBS buffer (pH 7.4), was incubated for 5 h at 30 °C. The control group consisted of 500 μM PAA. All samples were analyzed on a 1260 Infinity II gel permeation chromatography (GPC) system (Agilent, Santa Clara, CA, USA) with UltrahydrogelTM120, TM250, and TM500 columns (7.8 × 300 mm, Waters, Milford, MA, USA). The mobile phase was 0.1 M NaNO_3_ with a flow rate of 1 mL min^−1^ and a column temperature of 40 °C. The MW values of PAA in the control and treatment groups were obtained to observe the trend of MW changes of PAA before and after degradation. 

### 3.6. Enzyme Kinetic Analysis

To measure the Michaelis–Menten kinetic constants, the concentration of NADPH was varied while the concentrations of monooxygenase PCX02514 and PAA were fixed. A 2 mL mixture containing 1 mM PAA, 28 µM monooxygenase PCX02514, and 1 × PBS (pH 7.4) was premixed, and different concentrations of NADPH (10, 25, 50, 75, 100, 250, 500, and 1000 µM) were added to initiate the reaction. The mixture was then incubated for 5 h at 30 °C. In the control group, inactivated monooxygenase PCX02514 was added, and the other conditions remained unchanged. The absorbance of the reaction mixture at 340 nm was monitored using an optical microplate spectrophotometer (Tecan, Shanghai, China), with ΔA_340_ and the extinction coefficient of NADPH (6220 M^−1^ cm^−1^) used to calculate the rates of the reactions. The initial reaction rates against substrate concentrations were used to fit the Michaelis–Menten curve in GraphPad Prism software (https://www.graphpad.com/ (Version number: 9.5)).

### 3.7. Isothermal Titration Calorimetry

The calorimetric experiment was performed with a MALVERN PEAQ-ITC (Malvern, Great Malvern, UK). In the protein–ligand system, monooxygenase (PCX02514, 40 μM) was placed in the syringe, PAA (120 μM) was placed in the sample cell, and 1 × PBS buffer (pH 7.4, containing 100 μM NADPH) was placed in the reference cell. A typical experiment consisted of 13 injections: 0.4 μL for the first titration and 3 μL for each subsequent titration. The time interval between injections was 60 s, the agitator rotation speed was set to 750 rpm, and the binding of this system was monitored at 25 °C. For the blank control, 1 × PBS buffer (pH 7.4, containing 100 μM NADPH) was titrated with monooxygenase PCX02514 at 25 °C to deduct the dilution heat of monooxygenase PCX02514. Data with the background enthalpy subtracted were analyzed using the “one set of sites” model with MicroCal PEAQ-ITC Analysis Software (Version number: 1.41).

### 3.8. Crystallization, Data Collection, and Structure Determination

The purified monooxygenase PCX02514 was concentrated in 8 mg mL^−1^ and 15 mg mL^−1^ in a buffer containing 50 mM Tris-HCl (pH 8.0) and 150 mM NaCl. Initial screening of monooxygenase PCX02514 crystals was performed in a 96-well format using the sitting-drop vapor-diffusion method. Briefly, 1 μL of the protein solution (8 or 15 mg mL^−1^) was mixed with 1 μL of well solution and equilibrated over 100 μL of well solution. The screens were set up at 289 K using various crystal screening kits, including Index^TM^, Crystal Screen^TM^, Crystal Screen 2^TM^, PEG/Ion Screen^TM^, PEG/Ion 2 Screen^TM^ (HAMPTON RESEARCH, Aliso Viejo, CA, USA), and Wizard Classic 1–4 tubes (Rigaku, Tokyo, Japan). The optimal crystallization condition for monooxygenase PCX02514 was 1.0 M ammonium sulfate, 0.1 M HEPES pH 7.0, and 0.5% *w/v* PEG 8000. A single crystal was picked with a MicroLoop and flash-cooled in liquid nitrogen using a reservoir solution containing 20% glycerol as the cryoprotectant. The cryopreserved single crystal was mounted onto a local X-ray diffractor (BL18U1, wavelength of 0.9870 Å, Shanghai Synchrotron Radiation Facility, China) with 100 K nitrogen flow, and diffraction data were collected. The dataset was indexed, integrated, and scaled using the HKL3000 program suite [38]. Molecular replacement was performed using CCP4 [39] with a predicted AlphaFold [40] model as the search model. The structure was manually built with the modified experimental electron density in Coot [41] and further refined using Phenix [42] in iterative cycles. The details of the refinement of the structures determined through the employment of the molecular replacement methodology are comprehensively documented in Table 3. All structural figures were generated using PyMOL (https://www.pymol.org/ (Version number: 2.6.0a0)). The structure of monooxygenase PCX02514 was deposited in the PDB (https://www1.rcsb.org/ (accessed on 8 August 2024)) with accession number 8K74.

### 3.9. Molecular Docking of Monooxygenase PCX02514 and Substrates

The structures of the PAAs used in this study are shown in Figure S10. The LigPrep module (Schrödinger, LLC., New York, NY, USA) [43] was used to create minimal energy ligands. The crystal structure of monooxygenase PCX02514 was imported into the Protein Preparation Wizard of Schrödinger. Protein preparation was achieved by filling in missing loops or side chains, removing water molecules and unnecessary chains, optimizing structures of hydrogen bonds, and then minimizing energy. Molecular docking was conducted with the Glide module. A receptor grid was generated to search for the amino acids in the active pocket. After grid generation, the prepared conformations of the selected ligands were docked to the active sites utilizing the standard accuracy method. 

### 3.10. Site-Directed Mutagenesis and Enzyme Activity Measurement

Site-directed mutagenesis was used to introduce point mutations into the active sites of monooxygenase PCX02514, which were confirmed by sequencing. Briefly, PCR was performed in 25 μL reactions containing 50 ng of the pGEX-6P-1-orf02514 plasmid as the template, 0.4 μM forward and reverse primers (Table S5), 1 × Fast Alteration buffer, and 0.5 μL Fast Alteration DNA Polymerase (TIANGEN, Beijing, China). Reactions were performed on a T100 Thermal Cycler 10 (Bio-Rad, San Diego, CA, USA) with a primary denaturation at 94 °C for 5 min, followed by 25 cycles of 94 °C for 20 s, annealing at the specified temperature for 20 s, and 72 °C for 1.5 min, with a final extension for 10 min. The PCR mixtures were digested with *Dpn*I at 37 °C for 1 h to remove the template before being transformed into FDM-competent cells. Mutant monooxygenases were expressed and purified in a manner similar to that of the wild-type monooxygenase PCX02514. Subsequently, a 2 mL reaction mixture containing 500 μM PAA, 1 mM NADPH, and 14 μM monooxygenase mutant was incubated at 30 °C for 5 h. Inactivated enzymes were set as blank controls. The change in absorbance of NADPH at 340 nm was used to determine mutant enzyme activities, which were calculated and presented as a percentage of the wild-type enzyme activity.

### 3.11. Statistical Analysis

All experiments were repeated three times, including the control groups, and the results are represented as the mean ± standard deviation (X ± SD). The student’s *t*-test (SPSS Ver 20.0) was used for statistical analysis.

## 4. Conclusions

In this study, a novel monooxygenase PCX02514 was characterized and identified as a key enzyme in degrading PAA during PAM biodegradation. Biochemical experiments demonstrated that monooxygenase PCX02514 catalyzed the oxidation of PAA with the participation of NADPH and induced cleavage of the carbon chain. In addition, structural analysis indicated that the active pocket for PCX02514 was in a long cavity extending from the C-terminus of the TIM barrel to the protein surface and exhibited positive electrostatic potential, which was conducive to the migration of oxygen-negative ions into the active pocket. Furthermore, Arg10-Arg125-Ser186-Arg187-His253 in monooxygenase PCX02514 were proposed as potential active sites. The characterization of the molecular mechanism of the novel, efficient PAA-degrading monooxygenase in this study provides a theoretical basis for the development of efficient PAM biodegradation tools in the future. 

## Figures and Tables

**Table 1:** Enzyme kinetic parameters of monooxygenase PCX02514.

*K_M_* (μM)	V_m_ (μM s^−1^)	*k_cat_* (s^−1^)	*k_cat_*/*K_M_* (s^−1^ M^−1^)
158.9 ± 16.4	0.5376 ± 0.1284	0.00214 ± 0.000511	13.47

Note: Data are presented as mean ± standard deviation.

**Table 2:** Thermodynamic parameters of the interaction between PAA and monooxygenase PCX02514.

N (Sites)	KD (×10^−9^ M)	ΔH (kcal mol^−1^)	ΔS (cal mol^−1^ K^−1^)	ΔG (kcal mol^−1^)	−TΔS (kcal mol^−1^)
0.034 ± 0.0027	239 ± 15.7	−32.9 ± 5.42	−0.08 ± 0.013	−9.04 ± 1.63	23.9 ± 3.79

Note: Data are presented as mean ± standard deviation.

**Table 3:** Data collection and refinement statistics.

Protein	PCX02514
PDB accession	8K74
Data collection	
Beamline	BL18U1
Wavelength (Å)	0.9870
Space group	P 1 21 1
Cell dimensions	
*a*, *b*, *c* (Å)	73.23, 59.61, 79.72
α, β, γ (°)	90.00, 98.84, 90.00
Resolution (Å)	41.54–1.97(2.02–1.97) ^a^
No. observed reflections	44,451 (2618)
No. unique reflections	44,419 (2618)
*R*_merge (%)_	10.6 (68.0)
*I/σI*	8.2 (3.15)
Completeness (%)	92.7 (78.3)
Redundancy	1.0 (1.0)
CC1/2 (%)	0.0
Refinement	
Resolution (Å)	41.54–1.97
*R*_work_/*R*_free_	0.183/0.222
No. atoms	5201
Protein	4932
Ligand/ion	0
Water	269
*B-*factors	24.20
Protein	25.79
Ligand/ion	0
Water	27.76
R.m.s. deviations	
Bond lengths (Å)	0.007
Bond angles (°)	0.942
Ramachandran plot (%)	
Favored	97.83
Allowed	1.86
Disallowed	0.31

^a^ Values in parentheses are for the highest-resolution shell.

## Data Availability

The atomic coordinate and structure factor of the reported structure have been deposited in the PDB under accession code as follows: PCX02514, 8K74. The genomic sequence of *Klebsiella* sp. PCX has been uploaded to GenBank (https://www.ncbi.nlm.nih.gov (accessed on 8 August 2024)) with accession no. CP091527.1 and the associated BioProject and BioSample accession nos. were PRJNA595838 and SAMN13619442. The accession number of orf02514 was UKB70026.1. The original contributions presented in this study are included in the article/Supplementary Materials; further inquiries can be directed to the corresponding author.

## Supplementary Materials

The following supporting information can be downloaded at https://www.mdpi.com/article/10.3390/ijms25168871/s1.
