# Supplementary material for: Structural and Mechanistic Insights into a Novel Monooxygenase for Poly(acrylic acid) Biodegradation
Source: Int J Mol Sci. 2024 Aug 15;25(16):8871. doi: 10.3390/ijms25168871 (PMC11354265; doi:10.3390/ijms25168871)
Supplement: Supplementary file 1 [file ijms-25-08871-s001.zip › ijms-3137366-supplementary.pdf]

# Supporting Information

## Structural and mechanistic insights into a novel monooxygenase for poly(acrylic acid) biodegradation

Rui Feng[1], Juyi Zhao[1], Xiaochen Li[1], Sijun Dong[1], Dan Ma[1,*]

[1]College of Life Sciences, Hebei Basic Science Center for Biotic Interaction, Hebei University, Baoding, Hebei 071002, China

[*]Corresponding author. e-mail: danma@hbu.edu.cn

**Table Legends:**

Table S1. The molecular weights of substrates and products fitted with the obtained masses from HPLC-MS.

Table S2. The molecular weight distribution of PAA.

Table S3. Physicochemical properties of monooxygenase PCX02514.

Table S4. Primers for PCR.

Table S5. Mutagenesis oligonucleotides.

**Figure Legends:**

Figure S1. Gel-filtration elution and SDS-PAGE analyses of purified monooxygenase PCX02514.

Figure S2. Results of mass spectrometry.

Figure S3. X-ray diffraction pattern of monooxygenase PCX02514.

Figure S4. Structural comparison analyses of monooxygenase PCX02514 and bacterial luciferase family members.

Figure S5. Two-dimensional plots of the interactions between monooxygenase PCX02514 and PAAs.

Figure S6. Comparison of the binding pockets of 1RHC and PCX02514.

Figure S7. Structural conservation analysis of monooxygenase PCX02514.

Figure S8. Analysis of the electrostatic potential of monooxygenase PCX02514.

Figure S9. SDS-PAGE analysis of purified mutants.

Figure S10. Chemical structures of PAAs.

**Table S1.** The molecular weights of substrates and products fitted with the obtained masses from HPLC-MS.

| Reaction | Name | Molecular weight | Molecular formula | *m/z* [M-H]$^-$ |
|---|---|---|---|---|
| 1 | Substrate | 360.31 | $C_{15}H_{20}O_{10}$ | 359.3127 |
| | Product 1 | 216.19 | $C_9H_{12}O_6$ | 215.1880 |
| | Product 2 | 162.14 | $C_6H_{10}O_5$ | 161.1370 |
| 2 | Substrate | 432.38 | $C_{18}H_{24}O_{12}$ | 431.3780 |
| | Product 1 | 288.25 | $C_{12}H_{16}O_8$ | 287.2480 |
| | Product 2 | 162.14 | $C_6H_{10}O_5$ | 161.1370 |
| 3 | Substrate | 648.56 | $C_{27}H_{36}O_{18}$ | 647.5630 |
| | Product 1 | 504.44 | $C_{21}H_{28}O_{14}$ | 503.4410 |
| | Product 2 | 162.14 | $C_6H_{10}O_5$ | 161.1370 |

**Table S2.** The molecular weight distribution of PAA.

| Sample code | $M_n$ (g mol$^{-1}$) | $M_w$ (g mol$^{-1}$) | $M_z$ (g mol$^{-1}$) | $M_{z+1}$ (g mol$^{-1}$) | $M_w/M_n$ (-) |
|---|---|---|---|---|---|
| C1 | 1554 | 1576 | 1598 | 1618 | 1.014 |
| C2 | 1620 | 1642 | 1663 | 1684 | 1.013 |
| C3 | 1575 | 1587 | 1598 | 1607 | 1.021 |
| T1 | 1016 | 1033 | 1049 | 1065 | 1.017 |
| T2 | 1043 | 1058 | 1073 | 1087 | 1.014 |
| T3 | 1063 | 1080 | 1096 | 1113 | 1.016 |

Note: $M_n$: number average molecular weight, $M_w$: weight average molecular weight, $M_z$: z-average molecular weight, $M_{z+1}$: z+1 average molecular weight.

**Table S3.** Physicochemical properties of monooxygenase PCX02514.

| Protein | Isoelectric point (PI) | Molecular weight (kDa) | Extinction coefficient |
|---|---|---|---|
| PCX02514 | 6.21 | 35.77 | 0.628 |

**Table S4.** Primers for PCR.

| Gene name | Primer sequences (5'-3') | Restriction sites |
|---|---|---|
| orf02514 | CCGGAATTCATGCCTAAACGCCTCG | *Eco*RI |
| | CCGCTCGAGTTATCTGATGTGGGGGGC | *Xho*I |

Note: The underlined bases indicate protective bases, and the yellow highlights indicate restriction sites.

**Table S5.** Mutagenesis oligonucleotides.

| Name | Primer sequences (5'-3') |
|---|---|
| R10A | TAAACGCCTCGGCTTTTTACCGCTCTGCTCGATCAGGGCTC |
| | GAGCCCTGATCGAGCAGAGCGGTAAAAAGCCGAGGCGTTTA |
| R125A | CGGCCTGACCAGCGAGCAGGCCGGAGCGGTGTTTGCTGAC |
| | GTCAGCAAACACCGCTCCGGCCTGCTCGCTGGTCAGGCCG |
| S186A | CGGGACACGGGCTGATGCTGGCTCGCACGCAGCCCCGGCCGC |
| | GCGGCCGGGGCTGCGTGCGAGCCAGCATCAGCCCGTGTCCCG |
| R187A | CGGGACACGGGCTGATGCTGTCCGCCACGCAGCCCCGGCCGC |
| | GCGGCCGGGGCTGCGTGGCGGACAGCATCAGCCCGTGTCCCG |
| H253A | CGCAAACAGGCGGCACAGGCCCGGGAAGCGGGTCATCGGA |
| | TCCGATGACCCGCTTCCCGGGCCTGTGCCGCCTGTTTGCG |

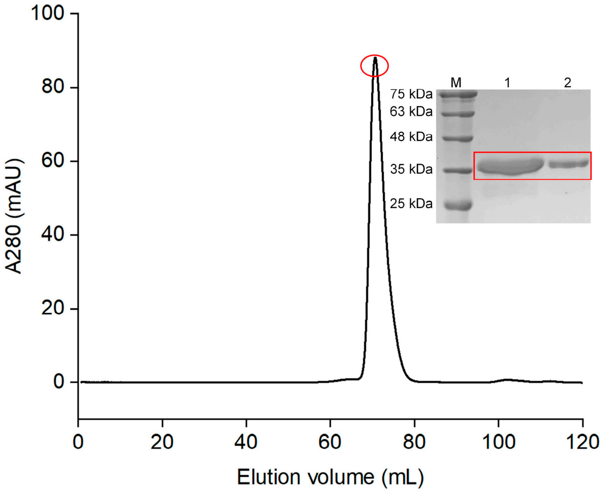


**Figure S1. Gel-filtration elution and SDS-PAGE analyses of purified monooxygenase PCX02514.** The chromatogram represented the elution profile of monooxygenase PCX02514 by gel-filtration column HiLoad 16/600 Superdex 200 prep grade. 12% SDS-PAGE gel with lane M: Protein molecular weight ladders; and lane 1-2: Purified monooxygenase PCX02514.

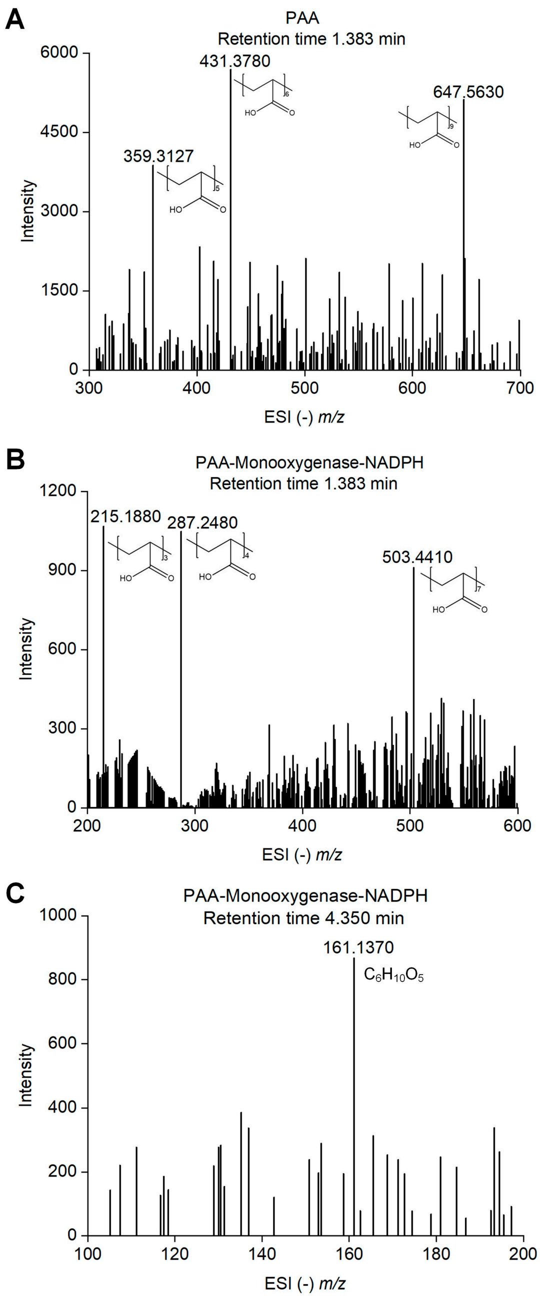


**Figure S2. Results of mass spectrometry.** (**A**) The ESI (-) *m/z* spectrum of the substrate peak. (**B**) The ESI (-) *m/z* spectrum of the product 1 peak. (**C**) The ESI (-) *m/z* spectrum of the product 2 peak.

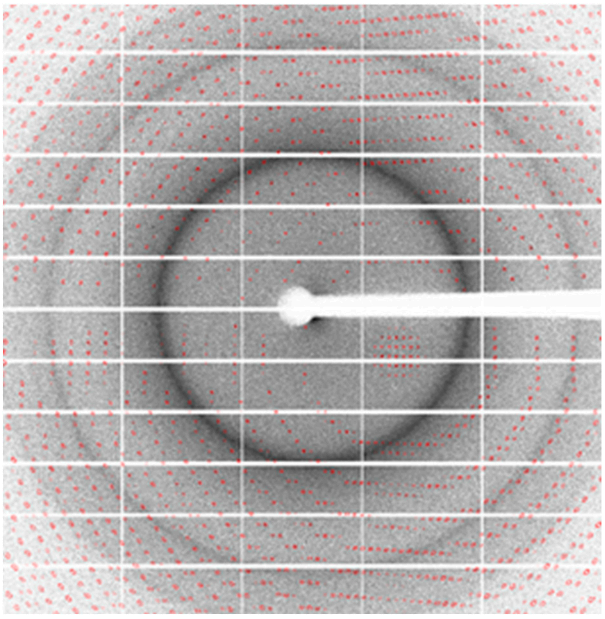

**Figure S3. X-ray diffraction pattern of monooxygenase PCX02514.**

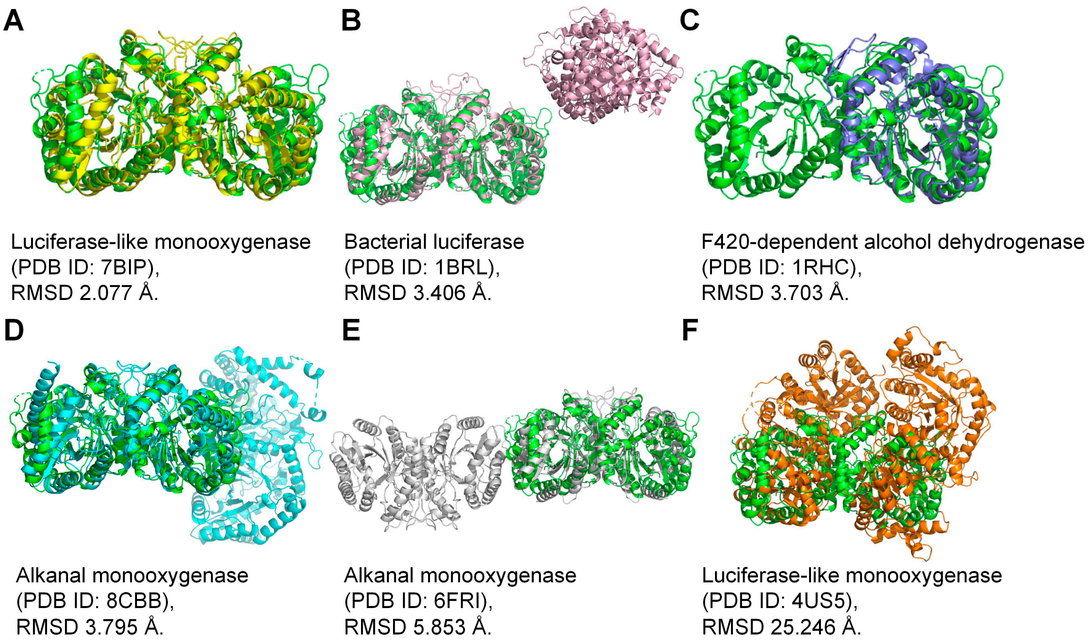


**Figure S4. Structural comparison analyses of monooxygenase PCX02514 and bacterial luciferase family members.**

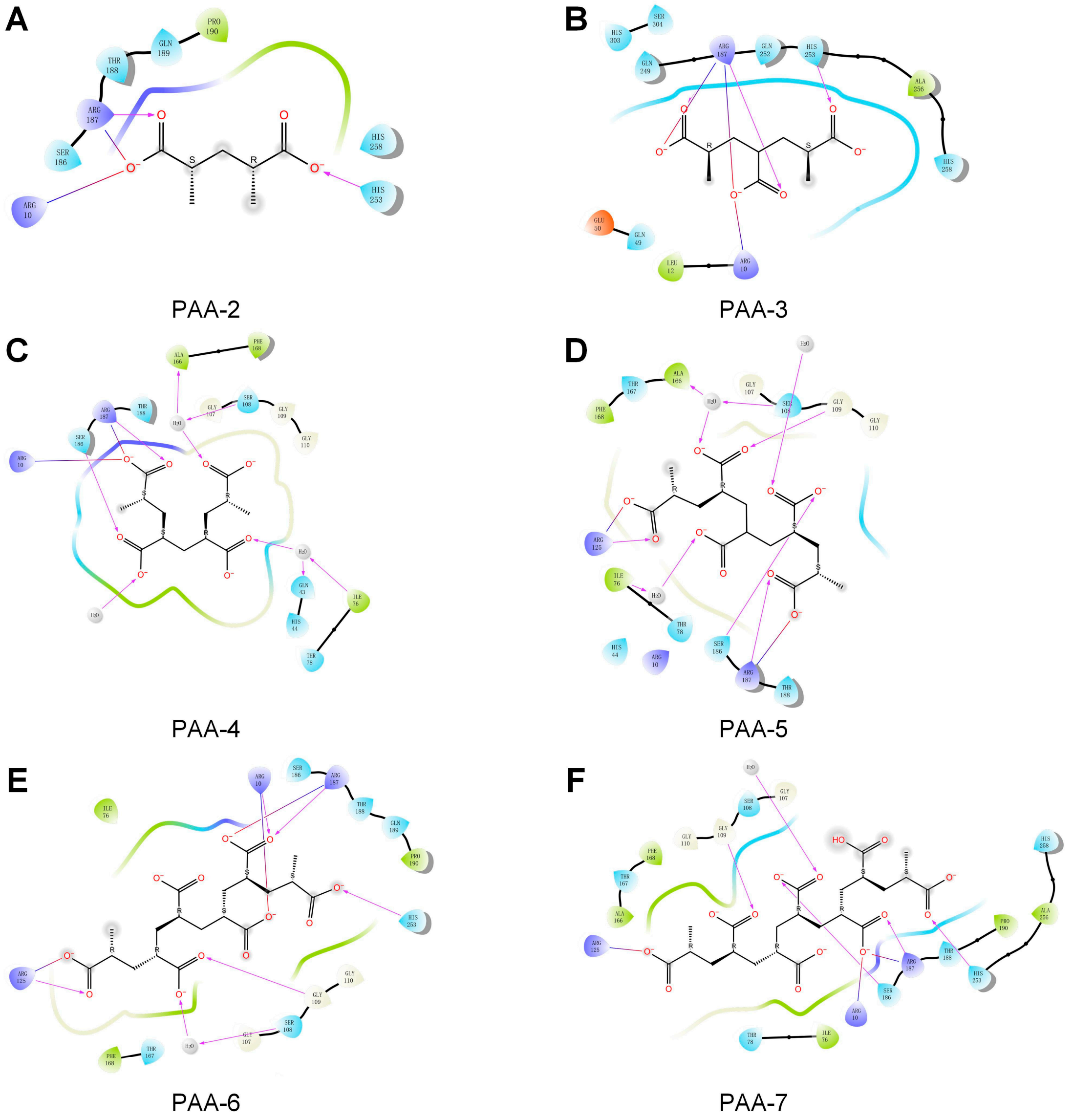


**Figure S5. Two-dimensional plots of the interactions between monooxygenase PCX02514 and PAAs.**

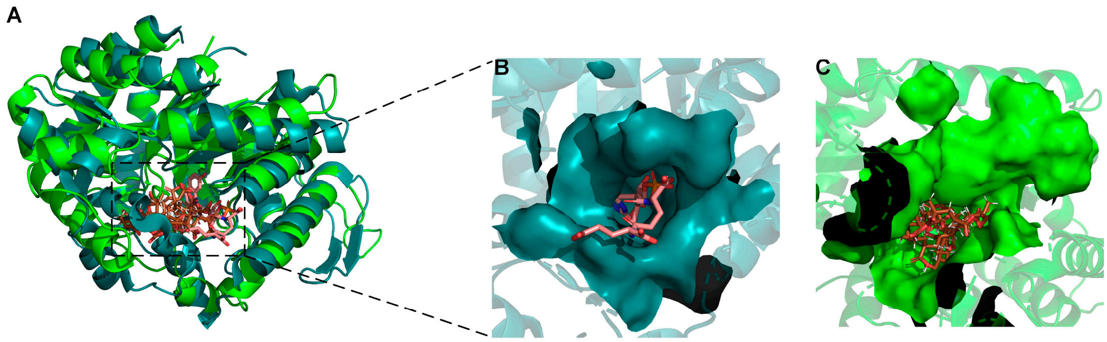


**Figure S6. Comparison of the binding pockets of 1RHC and PCX02514.** (**A**) Structural alignment of 1RHC and PCX02514. 1RHC was displayed in deepteal, and PCX02514 was displayed in green. (**B**) Enlarged view of the binding pocket of 1RHC. (**C**) Enlarged view of the binding pocket of PCX02514.

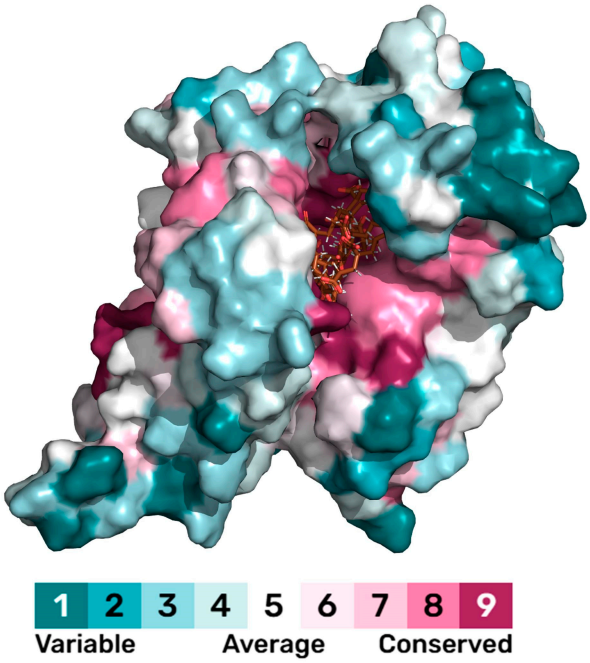


**Figure S7. Structural conservation analysis of monooxygenase PCX02514.** The results of structural conservation analysis were conducted using the ConSurf Web Server tool. The structural conservation files were visualized using PyMOL software.

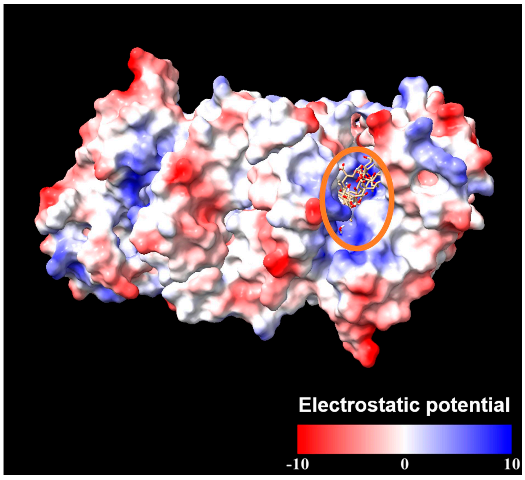


**Figure S8. Analysis of the electrostatic potential of monooxygenase PCX02514.**

The result of electrostatic potential was output by ChimeraX software.

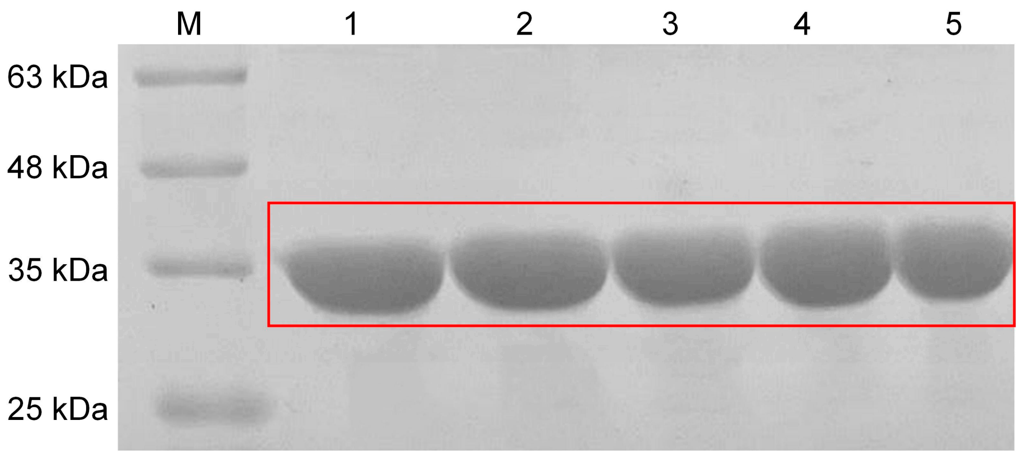


**Figure S9. SDS-PAGE analysis of purified mutants.** 12% SDS-PAGE gel with lane M: Protein molecular weight ladders; and lane 1-5: R10A, R125A, S186A, R187A, and H253A.

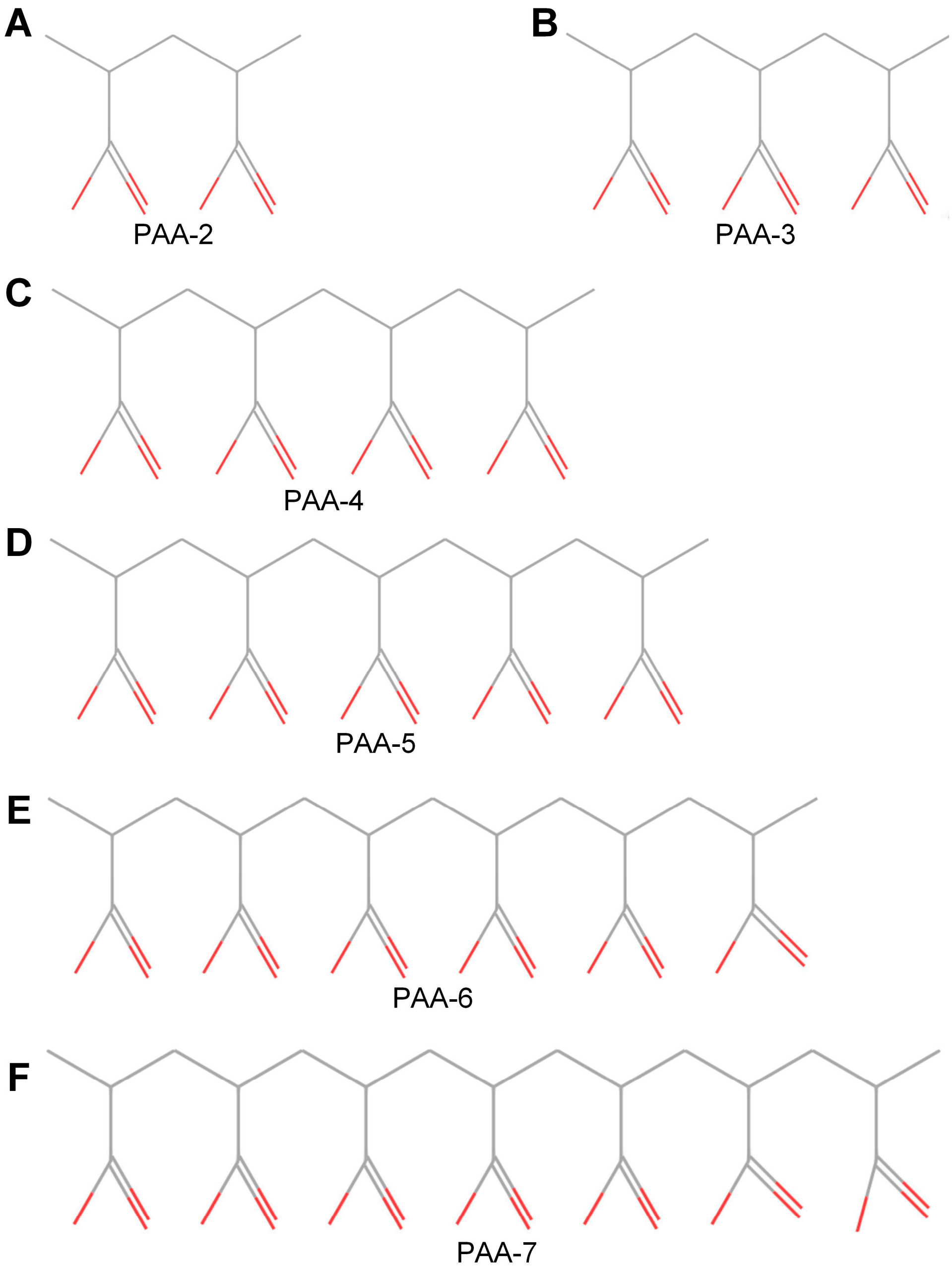


**Figure S10. Chemical structures of PAAs.** (**A**) PAA-2; (**B**) PAA-3; (**C**) PAA-4; (**D**) PAA-5; (**E**) PAA-6; (**F**) PAA-7.
